# Editorial: Regulatory immune cells in organ transplantation

**DOI:** 10.3389/fimmu.2024.1383563

**Published:** 2024-02-20

**Authors:** Hongxuan Ma, Jiajia Sun, Xiaohu Li, Yongsheng Luo, Jia Liu, Jinfeng Li

**Affiliations:** ^1^ Kidney Transplantation Unit, The First Affiliated Hospital of Zhengzhou University, Zhengzhou, China; ^2^ Dietetics Teaching and Research Section, Henan Medical College, Xinzheng, China

**Keywords:** regulatory immune cells, organ transplantation, tolerance, immunosuppression, immune response

Organ transplantation serves as a critical treatment for organ failure, facing a substantial challenge in maintaining long-term graft function due to immune-induced rejection. The pursuit of immune tolerance is imperative to liberate patients from reliance on immunosuppressive drugs. Regulatory immune cells were reported to contribute to immune homeostasis reconstruction and the induction of immune tolerance by restricting excessive immune responses. However, the mechanisms and effects of various immune cell subsets with immunomodulatory functions such as regulatory T cells (Tregs) in transplantation and tolerance induction remain incompletely understood (1). This Research Topic highlights the studies on regulatory immune cells in organ transplantation presented in the Research Topic to clarify the mechanism of immunoregulation and explore new therapeutic approaches.

Jin et al. developed a noninvasive technique for quantifying granzyme B (GzB) in cardiac allograft rejection using targeted contrast-enhanced ultrasound imaging. The study offered a safer alternative to endomyocardial biopsy which is regarded as gold standard by using microbubbles conjugated with anti-GzB antibodies to detect and quantify GzB expression. This approach effectively distinguished the difference between allogeneic (subject to rejection) and isogeneic (not subject to rejection) transplanted hearts based on GzB levels, indicating the potential as a novel tool for early detection of acute cardiac transplant rejection.

Poznansky et al. shed light on B-regulatory cells (Bregs), a subset of B-cells with tolerogenic functions. Bregs played a pivotal role in fostering graft acceptance within a tolerogenic milieu by secreting anti-inflammatory or tolerogenic cytokines. The study proposed a three-prong strategy, leveraging the complexity of TNF-α properties and engaging TNFR2 and TLR pathways, as a potential therapeutic approach to overcoming transplant rejection and promoting graft tolerance. LeGuern and Markmann provided a comprehensive overview of Tregs and their role as either permanent or temporary suppressors of immunity. By challenging the conventional understanding of Treg function, this study opened new avenues for developing therapies aimed at inducing transplant tolerance, emphasizing the nuanced and context-dependent nature of Treg-mediated immune regulation. Meanwhile, Cheng et al. reported the role of DNA-dependent activator of IFN regulatory factors (DAI) in alloimmune response after transplantation by targeting dendritic cells (DCs). The study reported that inhibition of the DAI led to reduced expression of co-stimulatory molecules and MHC-II on DCs and increased phagocytic ability, which promoted Treg differentiation and further highlighted a potential therapeutic strategy for improving transplant outcomes. Ali et al. explored the association between Human Leukocyte Antigen (HLA) class II alleles and rheumatoid arthritis risk, which offered a glimpse into the genetic underpinnings that may influence immune responses in a broader context including transplantation.

Against the backdrop of the COVID-19 pandemic, Rendina et al. delved into the immune response of solid organ transplant recipients. Specifically, the liver transplant recipients exhibit a similar risk of SARS-CoV-2 infection, mortality, and lethality as the general population, which challenged previous assumptions and possibly was may be linked to the grafted liver’s ability to induce immunotolerance.

Immunosuppression improves the organ transplant survival but increases susceptibility to fungal infections. Elalouf et al. addressed the delicate balance between immune responses and fungal infections in transplant recipients. Acknowledging the heightened susceptibility to fungal infections in immunosuppressed patients, the review emphasized early diagnosis and exploresd potential immuno-modulating treatments that leveraged innate immune responses. The integration of single-cell technologies with machine learning emerged as a promising avenue for biomarker discovery. Hu et al. brought attention to posttransplant lymphoproliferative disorders (PTLDs), a serious complication following solid organ transplantation (2). The case study of a lung transplant recipient with EBV-associated PTLD underscored the challenges in determining therapeutic intervention thresholds and the importance of monitoring EBV-DNA load. Meanwhile, the study emphasized the urgency for early diagnosis to enhance the likelihood of successful treatment.

This Research Topic illuminated the current state of research in the field of organ transplantation immunology by weaving together the findings from these studies. We underscored regulatory immune cells importance in immune tolerance and assessed their potential in diagnosis and therapy. Thus, the Research Topic unravelled the complexities of the immune system in organ transplantation and set a framework for future research directions that which devoted to improve patient outcomes and graft longevity.

## Author contributions

HM: Writing – original draft, Writing – review & editing. JS: Writing – original draft, Writing – review & editing. XL: Writing – original draft, Writing – review & editing. YL: Writing – original draft, Writing – review & editing, Conceptualization. JL: Conceptualization, Writing – original draft, Writing – review & editing. JFL: Conceptualization, Writing – original draft, Writing – review & editing.
